# Training shortens search times in children with visual impairment accompanied by nystagmus

**DOI:** 10.3389/fpsyg.2014.00988

**Published:** 2014-09-12

**Authors:** Bianca Huurneman, F. Nienke Boonstra

**Affiliations:** ^1^Department Cognitive Neuroscience, Donders Institute for Brain, Cognition and Behaviour, Radboud University Nijmegen Medical CentreNijmegen, Netherlands; ^2^Bartiméus, Institute for the Visually ImpairedZeist, Netherlands

**Keywords:** perceptual learning, fixation duration, visual impairment, visual search

## Abstract

Perceptual learning (PL) can improve near visual acuity (NVA) in 4–9 year old children with visual impairment (VI). However, the mechanisms underlying improved NVA are unknown. The present study compares feature search and oculomotor measures in 4–9 year old children with VI accompanied by nystagmus (VI+nys [*n* = 33]) and children with normal vision (NV [*n* = 29]). Children in the VI+nys group were divided into three training groups: an experimental PL group, a control PL group, and a magnifier group. They were seen before (baseline) and after 6 weeks of training. Children with NV were only seen at baseline. The feature search task entailed finding a target E among distractor E's (pointing right) with element spacing varied in four steps: 0.04°, 0.5°, 1°, and 2°. At baseline, children with VI+nys showed longer search times, shorter fixation durations, and larger saccade amplitudes than children with NV. After training, all training groups showed shorter search times. Only the experimental PL group showed prolonged fixation duration after training at 0.5° and 2° spacing, *p's* respectively 0.033 and 0.021. Prolonged fixation duration was associated with reduced crowding and improved crowded NVA. One of the mechanisms underlying improved crowded NVA after PL in children with VI+nys seems to be prolonged fixation duration.

## Introduction

Evidence is accumulating that perceptual learning (PL) can have a beneficial impact on a range of visual perceptual skills, from contrast sensitivity (Polat et al., 2009) to visual acuity (Hussain et al., 2012) and depth perception (Uka et al., 2012). However, the mechanisms underlying PL often remain unclear (Fiorentini and Berardi, 1980). Fiorentini rightfully questioned: “Where does this learning occur?” A review on the neural basis of PL reported that training has no effect on, or only weakly alters, sensitivity of neurons in early visual areas, but instead constitutes to long-term changes in high level decision making stages that read out sensory signals (Kumano and Uka, 2013). We recently demonstrated that PL not only induces specific learning effects on the trained task in children with visual impairment (VI), but also transfers to improvements in near visual acuity (NVA) (Huurneman et al., 2013). The majority of children involved in our intervention study showed nystagmus (33 out of 45). An important question is whether NVA improvements and reduced crowding ratios after PL are related to oculomotor changes in children with nystagmus. The present study investigates the influence of PL on feature search performance and oculomotor measures.

Our study was not the first to report improvements in NVA after PL. Two earlier studies presented NVA improvements in individuals with myopia (Durrie and McMinn, 2007), and individuals with presbyopia (Polat et al., 2012). The underlying mechanism thought to be responsible for the NVA improvements was more efficient and effective neural processing, which enhances the image quality by compensating for blurred retinal images due to optical defocus caused by a low degree of myopia or presbyopia (Durrie and McMinn, 2007; Polat et al., 2012). The authors found no changes in optical performance of the eye, i.e., accommodation, pupil, size or depth of focus that could explain the observed NVA improvements (Polat et al., 2012). This remarkable finding that NVA can be improved after PL in individuals with substantial vision loss due to aging, myopia, or VI deserves further investigation into the underlying mechanisms.

Nystagmus, i.e., involuntary ocular oscillations, can be the sole cause of reduced visual acuity (as in idiopathic infantile nystagmus syndrome), but can also be associated with congenital visual abnormalities such as albinism, congenital cataract or optic atrophy (Abadi and Bjerre, 2002). Nystagmus characteristics are usually captured in the following parameters: amplitude, frequency, intensity (i.e., amplitude × frequency), foveation characteristics and waveform types (Kumar et al., 2011). Longer foveation periods, i.e., the time during which a stable foveal image is present, are related to better visual acuity (Dell'osso and Daroff, 1975; Abadi and Worfolk, 1989; Bedell and Currie, 1993). Subjects with infantile nystagmus syndrome (INS) show reduced nystagmus amplitudes and frequencies when target size is at visual threshold, which illustrates that increased visual task demand can have a favorable impact on the nystagmus waveform (Wiggins et al., 2007). Others found that the foveation periods in subjects with INS are unrelated to optotype size (Tkalcevic and Abel, 2005). In contrast, task-induced stress and motivation appear to decrease foveation periods and increase nystagmus amplitude and intensity (Cham et al., 2008, 2013).

Neural operations underlying visual cognition take place mostly during fixations, since visual signal processing is limited during the execution of saccades, due to saccadic suppression, and therefore the bulk of visual analysis occurs during fixation (Volkmann, 1962; Volkmann et al., 1968). The nystagmus parameter that correlates best with visual acuity is fixation duration (Sheth et al., 1995; Abadi and Bjerre, 2002). Mean fixation durations typically vary between 200 and 500 ms in individuals with NV (Jacobs, 1986), but have been mentioned to lie between 20 and 150 ms (or as much as 400 ms) in individuals with INS (Sheth et al., 1995). Characteristics of infantile nystagmus are amplitudes between 0.3 and 15.7°, and frequencies have been reported to range between 0.5 and 10 Hz (Abadi and Bjerre, 2002). Peak velocities of the eye range between 20 and 180°/s (Abadi and Worfolk, 1989). Some report that nystagmus cannot be diminished voluntary, and attempts to fixate only counteract by exacerbating the nystagmus (Sheth et al., 1995). In adults with normal vision, adjustment of fixation duration is determined by the difficulty of previously presented trials (McPeek, 1995; Hooge and Erkelens, 1998).

Three training tasks were used in this study: a crowded/experimental magnifier training, a crowded/experimental PL training, and an uncrowded/control PL training. The two experimental training tasks were designed with the aim to reduce crowding effects in children with VI. The rationale behind the design of these two experimental training tasks is that training tasks with small element spacing would eventually result in an improved ability to identify objects in clutter.

In order to find out whether oculomotor parameters change after PL, we recorded eye movements during visual feature search before and after 6 weeks of training. Four hypotheses were evaluated. The first is that there are baseline differences in fixation duration between children with normal vision (NV) and children with VI accompanied by nystagmus (VI+nys). The second is that search times of children with VI+nys are longer than search times of children with NV. The third is that training induces shorter search times in all training groups. Finally, it is hypothesized that there are no changes in fixation duration after training in children with VI+nys.

## Materials and methods

### Participants

Thirty-three children with VI+nys and 29 children with NV participated. Inclusion criteria for both groups were: age between 4 and 9 years and a normal developmental level. Additional inclusion criteria for children with VI were: distance visual acuity (DVA) between 20/400 and 20/40, normal birth weight (at least 3000 grams), birth at term (at least 36 weeks), no perinatal complications, no additional impairments, and an intact visual field. Table 1 presents the average age and DVA of the children with VI and with NV. The supplemental Table presents clinical diagnosis and characteristics of the children in the VI+nys group. Informed consent was obtained from the parents of all children after explanation of the nature and possible consequences of the study. The local ethics committee approved the study before the assessments were conducted (CMO Arnhem-Nijmegen). The study was conducted in accordance with the Declaration of Helsinki (1969).

**Table 1:** **Characteristics of children with normal vision (NV) and children with visual impairment accompanied by nystagmus (VI+nys) with age presented in months and distance visual acuity (DVA) presented in logMAR notation for the crowded version of the *C*-test [mean, (SD)]**.

	**NV**			**VI+nys**		
	**M^a^**	**PLc^b^**	**PLu^c^**	**M**	**PLc**	**PLu**
*N*	9	10	10	9	15	9
Age	78.3 (18.1)	81.6 (16.3)	83.9 (16.9)	76.9 (13.3)	81.80 (19.0)	87.8 (18.2)
DVA	0.04 (0.16)	0.00 (0.10)	−0.04 (0.08)	0.82 (0.17)	0.77 (0.27)	0.74 (0.31)

a *M, magnifier group*.

b *PLc, perceptual Learning crowded group*.

c *PLu, perceptual learning uncrowded group*.

### Ophthalmological examination

All children underwent ophthalmological examination before the start of the experiment. Distance visual acuity was measured mono- and binocularly at 6 m with the tumbling E chart (Taylor, 1978) and the Landolt *C*-test at 5 m (Haase and Hohmann, 1982) under controlled lighting conditions. NVA was determined binocularly with the LH-version of the *C*-test (Huurneman et al., 2012) and the LH line 50% crowding chart (Hyvarinen et al., 1980) at 40 cm. The eye-to-chart distance during NVA measurements was monitored with a ruler. The LH-version of the *C*-test contains two chart versions with absolute spacing: a crowded chart with interoptotype spacing of 2.6′, and a single chart with interoptotype spacing of ≥30′ at 40 cm. Children were asked to identify the first five symbols in a row, which were pointed out with a pencil, and could progress to the next line if they correctly identified 3 or more out of the 5 symbols. If there were fewer than 5 symbols in a row, children could progress if they could correctly identify at least half of the symbols.

Visual field was estimated by using confrontational techniques. In case of retinal disease children were tested on central or peripheral scotomas with dynamic perimetry (Goldmann). Objective refraction was obtained after cycloplegia and if necessary spectacle correction was prescribed or changed before the experiment and training period started.

### Training task

Children were divided into one of three training groups matched on age and distance visual acuity (see Table 1). There were two experimental groups and one control group (Huurneman et al., 2013). The first experimental training paradigm was a visual search training in which children had to search for the smiley in a grid of E's sized 145 × 145 mm and an edge-to-edge element spacing of 0.3 mm (consistent with the interoptotype spacing of the crowded version of the C-test described above, which is 2.6′ or 0.04° at 40 cm). This group was called the PL crowded group (PLc group). When the children found the smiley, they had to follow the path of inversed E's (leftward pointing E's). The E's were initially sized 7.0 mm, consistent with 1.0 logMAR at 40 cm. If the children did not make any errors, they could proceed to training material with smaller E's (two subsequent steps: 0.5 logMAR [3.5 mm] and 0.25 logMAR [1.75 mm]). As can be imagined, this task puts a strong demand on oculomotor and on interference control, as it was inspired by the Eriksen flanker task (Eriksen and Eriksen, 1974). The control training task was essentially the same task, but element spacing was 3.6 mm instead of 0.3 mm (consistent with the interoptotype spacing of the uncrowded *C*-test, which is at least 30′ or 0.5° at 40 cm). Therefore, the control group was the PL uncrowded group (PLu group).

The third training paradigm was a magnifier training in which children had to search for the inversed Landolt C within a 191 mm long search strip consisting of Landolt C's sized 0.5 mm (or −0.10 logMAR) with element spacing of 0.3 mm (the M group). The optotypes were too small to inspect with a bare eye, so an electronic handheld magnifier was used to find the inversed C. A game element was included to provide feedback and engage the children. Each training consisted of a maximum of 12 trials and these trials were coupled to tiles that could be placed on answer boxes. If all tiles were placed correctly, they formed a pattern that matched with the pattern in the upper right corner of the page. During training, children worked at a self-chosen distance.

### Procedure

#### Training

The training tasks are described in the section Training Task. Children with NV were seen once as a reference group at baseline. For children with VI, this baseline performance counted as their pre-test score. Training started within 2 weeks after the pre-test. During the training, children with VI were seen 2× a week for a period of 6 weeks (a total of 12 training sessions). Each training session consisted of 30 min of practice on the training task described in the section above. Trainers visited children at their schools. Within 2 weeks after the last training session children performed the post-test.

#### Visual search task

**Figure 1:**
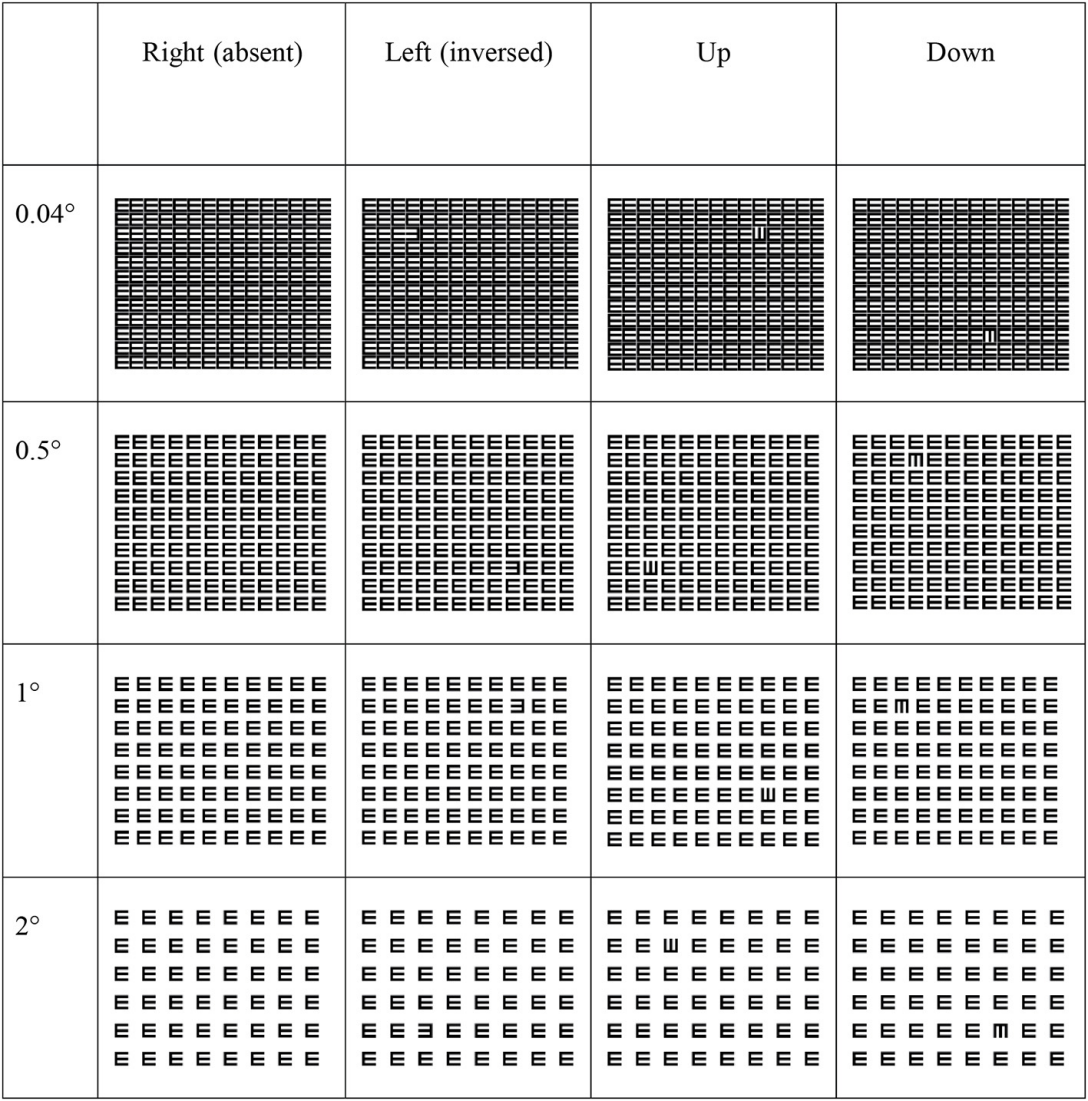
**Examples of the stimuli used in our experiment**.

Children sat at a distance of 60 cm from the monitor wearing their best available optical correction. Viewing was binocular. Sixteen trials were presented to the children (4 (orientation) × 4 (spacing), see Figure 1). Four practice trials preceded the experiment in order to familiarize children with the task. Trials were presented in a randomized order to avoid measuring influences of learning effects. The target subtended 2° × 2° and the stimulus was screen-wide (29° × 25°). The instruction was to identify the unique E or, in case the E was absent, press the right pointing E (target absent trial). The location of the E was randomly varied in each quadrant of the screen to make sure the child actively searched for the target. Before the trial commenced a fixation cross of 2° × 2° was presented for 500 ms after which an inter-trial-interval of 1000 ms followed. The influence of crowding was measured by manipulating spacing, with edge-to-edge element spacing of 0.04°, 0.5°, 1°, and 2°. There was no time limit for performing the search task. A new trial was presented after the child pressed the response button matching the target.

### Apparatus

Stimuli were presented on a 17″ monitor with integrated eye-trackers (Tobii T120, Tobii Corporation, Danderyd, Sweden). Stimulus presentation was driven by Delphi software. Eye movements were analyzed using MATLAB (MathWorks, Inc., Natick, MD). We did not fix the head positions of the children, because the Tobii has a large head movement box which means that it has a good ability to compensate for head movements (44 × 22 cm at 70 cm). Children stayed well within these limits, so head movements are not likely to cause data distortions. A rule was incorporated into the stimulus-presentation software, in order to assure that the children were seated at a proper viewing distance. When children came closer to the monitor than 60 cm, the stimulus disappeared from the screen, and reappeared if they were seated at 60 cm or more again. This rule was included to prevent children from reducing their viewing distance and to standardize our measurements. Eye movements were registered at a sampling rate of 60 Hz. Before the visual search task commenced a standard 5-point calibration procedure was performed. Fixations were detected offline and were defined as periods in which eye velocity remained below 50°/s for at least 50 ms. The velocity threshold was recalculated as 3.5× the standard deviation of the calculated noise of the eye velocity for each session. This recalculation is necessary, because of the diversity of the eye movements of our participants. For example, eye movement velocity might be seriously elevated in children with nystagmus and we might need a higher threshold than 50°/s to detect fixations. Data files with less than 35% valid data points were excluded from analysis.

### Statistical analysis

There were 2 main outcome categories: visual search performance measures (accuracy and search time) and oculomotor measures (number of fixations, fixation duration, and saccade amplitude). Accuracy measures were compared between children with NV and children with VI+nys with nonparametric tests (Mann–Whitney test and Friedman's test), because of skewed distributions and unequal variances. A correction for pairwise comparisons was used for the Friedman's test by reporting the adjusted *p*-value in which the K refers to the number of groups (*p*_adj_ = p^*^K (*K* − 1)/2).

Search time showed near-normal distribution and oculomotor measures were distributed normally. These measures were analyzed with a Repeated Measures ANOVA. Group was entered as the between-subjects factor and spacing as the within-subjects factor. Training effects in children in the VI+nys group were evaluated by conducting a 2 (pre-post) × 4 (spacing) Repeated Measures ANOVA. Training group was then entered as the between-subjects variable. In case of pre-post × training group interactions, we conducted paired *t*-tests for each training group to see under which condition changes in performance occurred. In case pre-post × spacing interactions were found, separate Repeated Measures ANOVA's were conducted to evaluate how spacing affected performance before and after training. *Post-hoc* tests were performed using the Bonferroni method to correct for multiple comparisons (familywise Type I errors). Alpha was set on 0.10 to enable the detection of trends in our data set and because of the small sample sizes.

## Results

### Group differences at baseline

**Figure 2:**
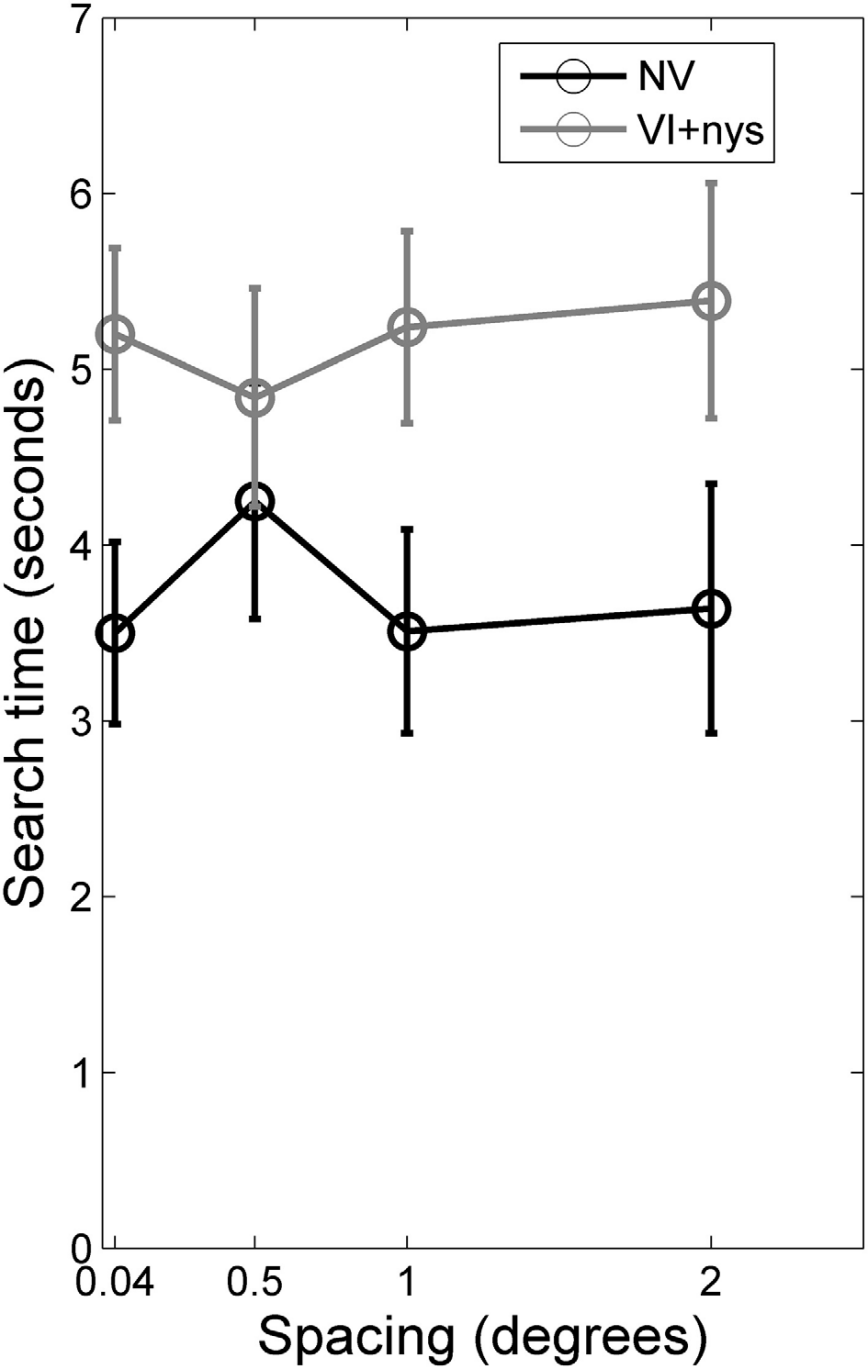
**Search times for children with normal vision (NV) and children with visual impairment accompanied by nystagmus (VI+nys)**. Children with NV showed shorter search times than children with VI+nys. Error bars indicate standard error of the mean (s.e.m.).

**Figure 3:**
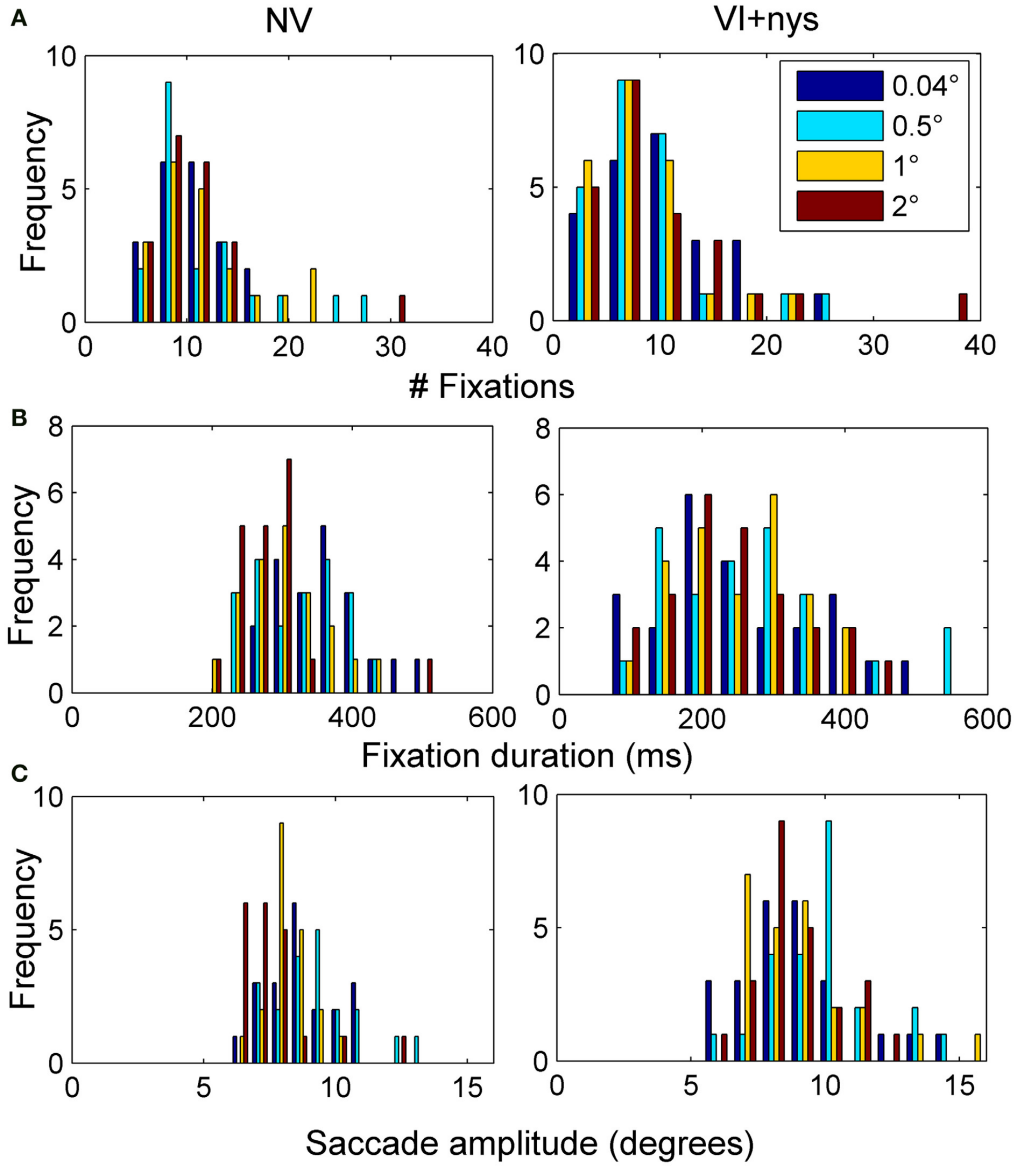
**(A)** Distribution of the number of fixations made by children with normal vision (NV) and children with visual impairment accompanied by nystagmus (VI+nys). **(B)** Distribution of fixation durations. **(C)** Distribution of saccade amplitudes.

**Figure 4:**
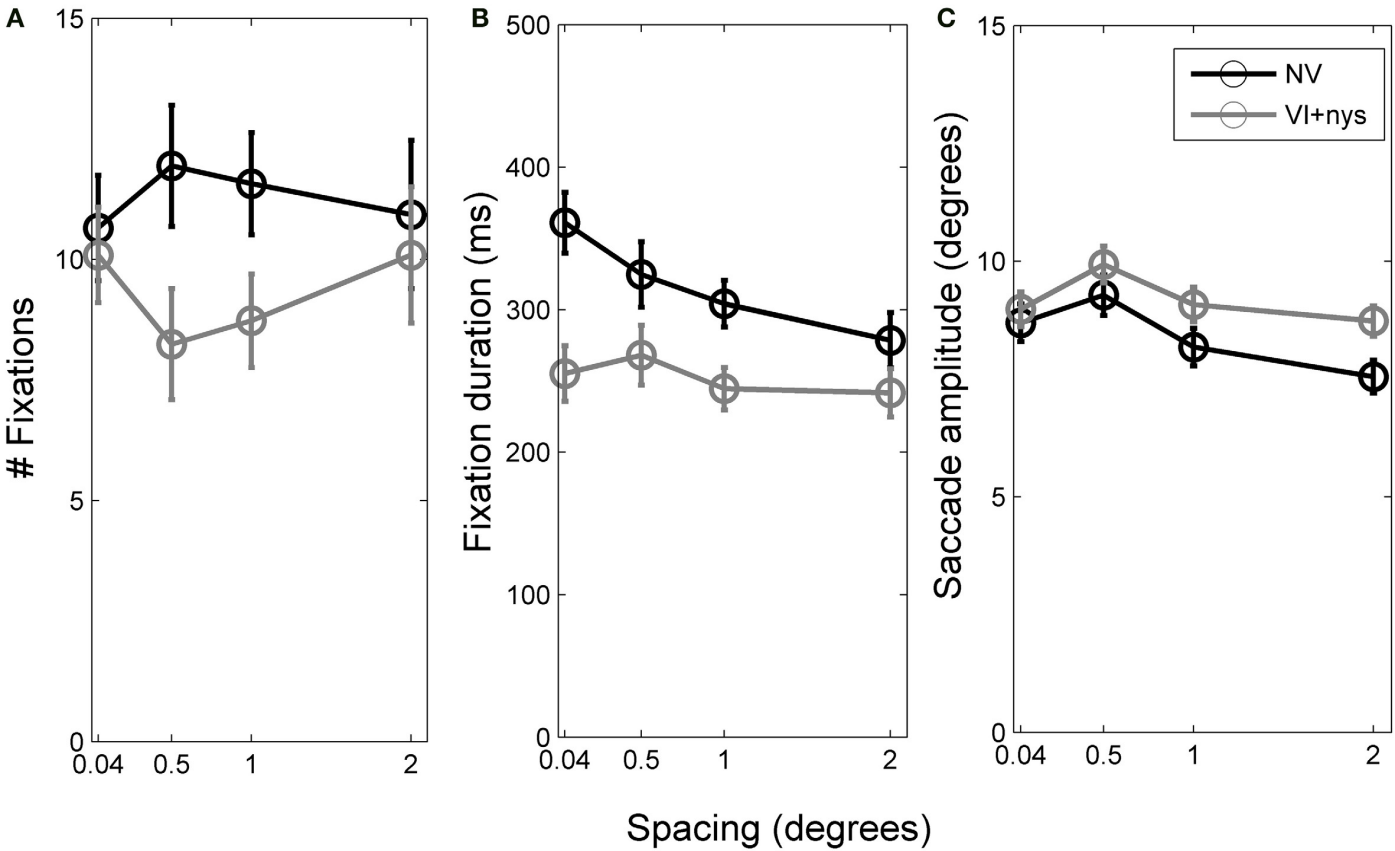
**(A)** Number of fixations per trial made by children with normal vision (NV) and children with visual impairment accompanied by nystagmus (VI+nys). **(B)** Fixation durations of children with NV and children with VI+nys. **(C)** Saccade amplitudes of children with NV and children with VI+nys. Error bars represent s.e.m.

Figure 2 presents group differences in search time at baseline, Figure 3 presents distributions of oculomotor measures, and Figure 4 presents group differences in oculomotor measures at baseline. Valid oculomotor measures were collected in 20 children with NV, and 24 children with VI+nys.

#### Performance measures

***Accuracy***. No group differences were found in accuracy (see Table 2). There was a main effect of spacing on accuracy in both groups [NV: χ^2^_(3)_ = 22.25, *p* < 0.001; VI+nys: χ^2^_(3)_ = 11.68, *p* = 0.009]. Children with NV showed lower accuracies at 0.04° and 0.5° (75%) than at 2° spacing (100%), resp. *p* = 0.026 and *p* = 0.005. Pairwise comparisons in the VI+nys group were non-significant (see Table 2).

**Table 2:** **Median accuracies and statistics for accuracy**.

**Spacing**	**Accuracy medians**		**Group difference [standardized test statistic (*z*)]**	**Within-subjects effects**	
	**NV (%)**	**VI+nys (%)**		**NV**	**VI+nys**
0.04°	75	75	*z* = 0.23, *p* = 0.821	χ^2^_(3)_ = 22.25	χ^2^_(3)_ = 11.68
0.5°	75	75	*z* = 0.96, *p* = 0.337	*p* < 0.001	*p* = 0.009
1°	75	100	*z* = 0.39, *p* = 0.694	0.04° < 2°^***^	*Post hoc* n.s.
2°	100	100	*z* = −0.88, *p* = 0.380	0.5°< 2°^**^	

^*^p < 0.1,

** *p < 0.05*,

*** *p < 0.01*.

***Search time***. There was a group difference in search time [*F*_(1, 60)_ = 3.71, *p* = 0.059, partial η^2^ = 0.06]. Children with NV showed shorter search times (3.7 s) than children with VI+nys (5.2 s; see Figure 2). No spacing effect or spacing × group interaction was found.

#### Oculomotor measures

***Number of fixations***. Groups did not differ with respect to the number of fixations made (*p* > 0.1, see Figure 4A). Children showed an average of 10.3 fixations (*SE* = 0.7). No spacing or interaction effect was found.

***Fixation duration***. There was a group × spacing interaction, *F*_(3, 126)_ = 2.57, *p* = 0.057, partial η^2^ = 0.06. At 0.04°, children with VI+nys showed shorter fixation durations (255 ms) than children with NV (361 ms), *F*_(1, 42)_ = 13.31, *p* = 0.001, partial η^2^ = 0.24. At 0.5°, children with VI+nys showed shorter fixation durations (268 ms) than children with NV (325 ms), *F*_(1, 42)_ = 3.34, *p* = 0.075, partial η^2^ = 0.07. At 1°, children in the VI+nys group showed shorter fixation durations (245 ms) than children in the NV group (304 ms), *F*_(1, 42)_ = 7.21, *p* = 0.010, partial η^2^ = 0.15. At the largest spacing (2°), no group differences were found. Children with NV adjusted their fixation durations to spacing, *F*_(3, 57)_ = 7.45, *p* < 0.001, partial η^2^ = 0.28. Children fixated longer at 0.04° than at 1° and 2° spacing, *p*'s 0.037 and 0.002 (see Figure 4B). Spacing did not affect fixation duration in children in the VI+nys group.

***Saccade amplitude***. Saccade amplitudes differed between groups, *F*_(1, 42)_ = 8.22, *p* = 0.006, partial η^2^ = 0.16. Children in the VI+nys group made larger saccades (9.2°) than children with NV (8.4°: see Figure 4C). Spacing influenced saccade amplitude, *F*_(3, 126)_ = 5.09, *p* = 0.002, partial η^2^ = 0.11. Saccade amplitudes were larger at 0.5° (9.3°) than at 2° (7.9°), *p* = 0.001. No interaction was found.

### Differences after 6 weeks training (VI children)

**Figure 5:**
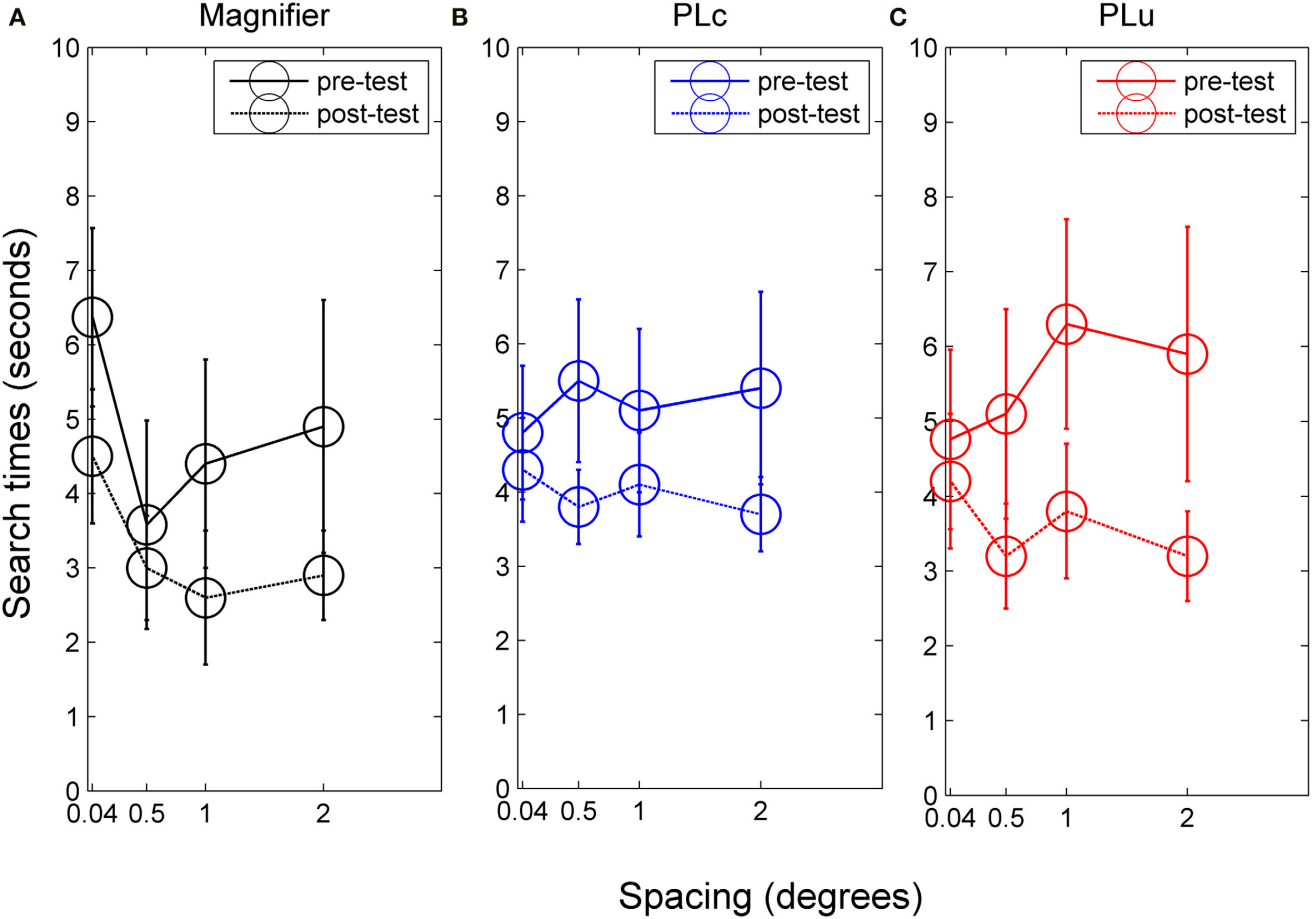
**Search times for (A) children in the magnifier group, (B) children in the PLc group before and after training, and (C) children in the PLu group before and after training**. Error bars represent s.e.m.

**Figure 6:**
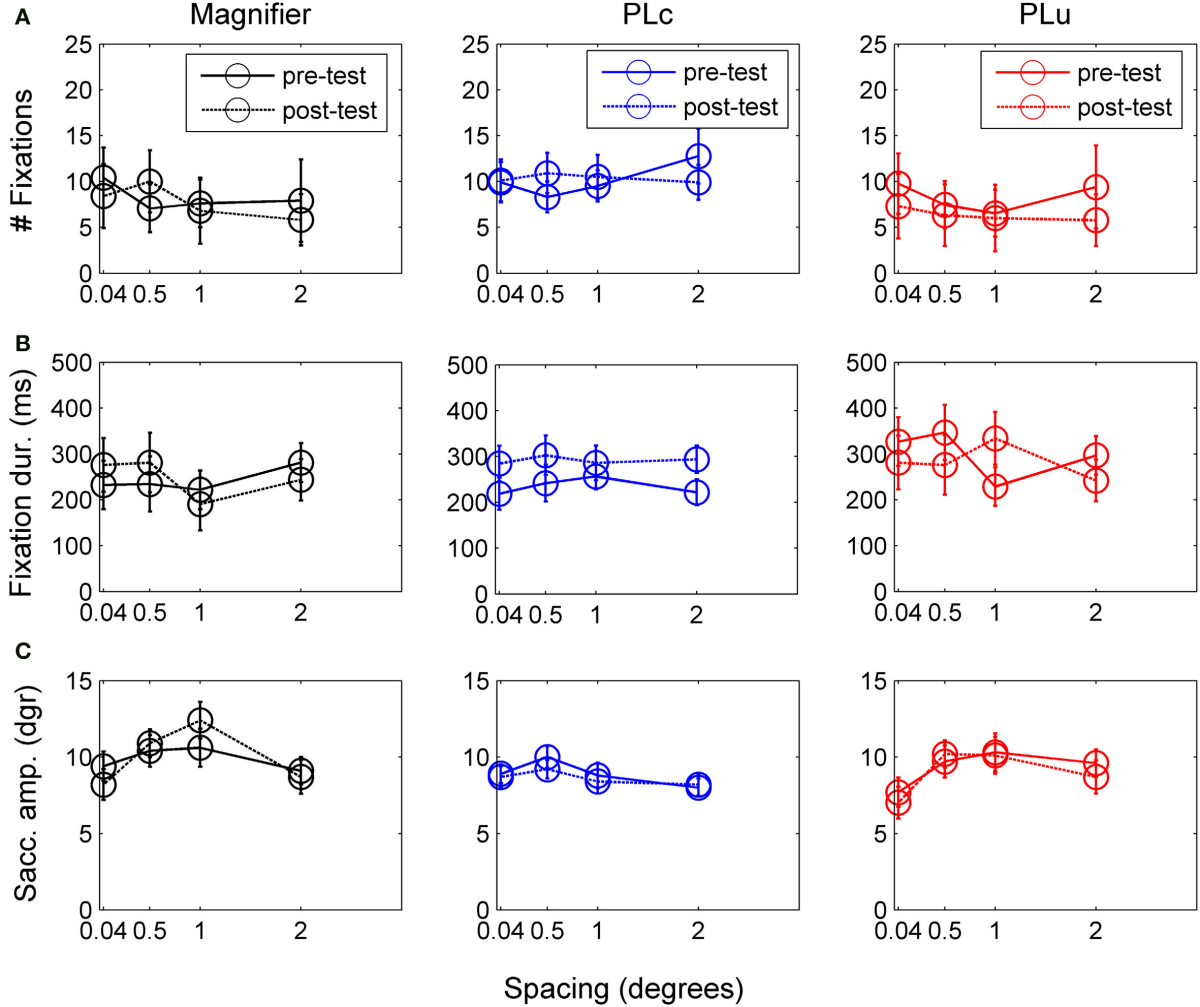
**(A)** Number of fixations before and after training. **(B)** Fixation duration before and after training. **(C)** Saccade amplitude before and after training. Error bars represent s.e.m.

We collected 17 valid eye movement recordings (magnifier, *n* = 4; PLc, *n* = 9; PLu, *n* = 4). Figure 5 displays search times and Figure 6 displays oculomotor measures before and after training. Supplemental Figures 1–3 display histograms with raw pre- and post-training oculomotor measures.

#### Performance measures

***Accuracy***. At 0.5°, the M and PLc group showed higher accuracies after training (median before training 75% and 100% after training: M: χ^2^_(1)_ = 5.00, *p* = 0.025; PLc: χ^2^_(1)_ = 5.00, *p* = 0.025). Children in the PLu group did not show improved accuracy; they already showed a median accuracy of 100% at pre-test. At 0.04°, 1° and 2°, none of the groups showed improved accuracy after training.

***Search time***. There was a spacing × training group interaction, *F*_(6, 90)_ = 2.14, *p* = 0.057, partial η^2^ = 0.13. In the M group, a training effect was found, *F*_(1, 24)_ = 10.31, *p* = 0.012, partial η^2^ = 0.56. Mean search time was 4.8 s before and 3.3 s after training (see Figure 5A). In addition, spacing influenced search times, *F*_(3, 24)_ = 3.78, *p* = 0.024, partial η^2^ = 0.32. However, *post-hoc* tests were not significant. No pre-post × spacing interaction was found. In the PLc group, we also found a training effect, *F*_(1, 42)_ = 4.99, *p* = 0.042, partial η^2^ = 0.26. Mean search times were 5.2 s before and 4.0 s after training (see Figure 5B). No spacing or pre-post × spacing interaction was found. A pre-post × spacing interaction did occur in the PLu group, *F*_(3, 24)_ = 2.37, *p* = 0.096, partial η^2^ = 0.23. Paired *t*-tests showed that children did not show reduced search times at 0.04°, but did show reduced search times at all other spacings [0.5°: *t*_(8)_ = 2.15, *p* = 0.064, 1°: *t*_(8)_ = 1.88, *p* = 0.097, 2°: *t*_(8)_ = 1.98, *p* = 0.083; see Figure 5C].

#### Oculomotor measures

***Number of fixations***. There was no main training effect on the number of fixations, but there was a pre-post × spacing interaction, *F*_(2, 14)_ = 3.83, *p* = 0.071, partial η^2^ = 0.22. Children made more fixations before (10.0) than after training at 2° (7.1), *t*_(16)_ = 2.23, *p* = 0.041. All other *t*-tests were non-significant.

***Fixation duration***. There was a three way pre-post × training group × spacing interaction, *F*_(1, 42)_ = 2.24, *p* = 0.058, partial η^2^ = 0.24. Paired *t*-tests were conducted for each spacing and training group to disentangle interaction effects. In the M group, no changes were found in fixation duration (see Figure 6B). In the PLc group, children fixated longer after training at 0.5° and 2° (at 0.5°: *t*_(8)_ = −2.58, *p* = 0.033; at 2°: *t*_(8)_ = −2.87, *p* = 0.021, see Figure 6B). In the PLu group, prolonged fixation duration was found at 1°, *t*_(3)_ = −2.49, *p* = 0.089 (see Figure 5B). No spacing or interactions were found in any of the training groups.

***Saccade amplitude***. There was a spacing × training group interaction, *F*_(6, 42)_ = 2.12, *p* = 0.071, partial η^2^ = 0.23. *Post-hoc* analysis showed that in the M-group, spacing affected saccade amplitude, *F*_(3, 9)_ = 4.21, *p* = 0.040, partial η^2^ = 0.58. Children tended to make larger saccades at 0.5° (10.4°) than 2° (8.7°), *p* = 0.055 (see Figure 6C). In the PLc group, no main or interaction effects were found. In the PLu group, spacing affected saccade amplitude, *F*_(3, 9)_ = 4.51, *p* = 0.034, partial η^2^ = 0.60. However, *post-hoc* tests were non-significant.

### Relations between acuity, fixation duration and performance measures

A correlational analysis was conducted to investigate the relations between changes in visual and oculomotor measures for children in the VI+nys group. As can be seen in Table 3, the increase in fixation duration was related to a reduction of the crowding ratio and improved crowded NVA, *r's* 0.65 and 0.52, *p's* 0.002 and 0.016.

**Table 3:** **Correlation matrix displaying the relations between visual and oculomotor measures**.

	**Gain logMAR single**	**Gain logMAR crowded**	**Fixation duration gain at 0.5°**	**Reduction search time at 0.5°**	**Improvement accuracy at 0.5°**
Gain logMAR crowded	0.61^*^				
Fixation duration gain at 0.5°	−0.41	0.52^*^			
Reduction search time at 0.5°	−0.05	0.52^*^	0.03		
Improvement accuracy at 0.5°	0.19	0.35	−0.03	0.14	
CR reduction	−0.77^**^	0.62^**^	0.65^**^	0.30	0.11

*CR reduction, crowding ratio reduction (CR pre-test–CR post-test)*.

* *p < 0.05*.

** *p < 0.01 (one-tailed p-test)*.

## Discussion

The goal of this study was to evaluate the effect of PL on visual feature search performance and oculomotor measures in children with VI+nys. Different from what we expected, we found evidence that oculomotor parameters in children in the VI+nys group can be altered after a 6-week period of PL. We only observed significant increases in fixation duration in the PL groups. In the next sections our findings are discussed in the light of the postulated hypotheses.

### Group differences in fixation duration between groups at baseline

Our first hypothesis was that there were group differences in fixation duration at baseline. Children with NV showed longer fixation durations (278–361 ms) than children in the VI+nys group (242–268 ms). This finding is in line with results from a previous study from our group which reported shorter fixation durations in children in the VI+nys group compared to children with NV during serial visual search (Huurneman et al., 2014). A second difference between groups occurred with regards to the adjustment of fixation duration to element spacing. Children with NV showed an adjustment of fixation duration to stimulus properties and fixated longer on stimuli when element spacing was smaller (346 ms at 0.04° and 292 ms at 2° spacing). This finding is consistent with oculomotor behavior observed in adults with NV (Vlaskamp et al., 2005; Vlaskamp and Hooge, 2006). Children in the VI+nys group showed no adjustment of fixation durations to element spacing, neither before or after 6 weeks of PL.

In addition to group differences in fixation duration, a group difference in saccade amplitude was found. Mean saccade amplitude of children in the NV group was smaller (8.4°) than the saccade amplitude of children in the VI+nys group (9.2°). Saccade amplitudes were calculated by measuring the distance from one fixation point to a following fixation point. The finding that children with VI+nys make larger saccades than those with NV is a replication of what was reported in an earlier study (Huurneman et al., 2014). We did not control for the influence of cases when children were looking away from the screen during search. If the child looks off screen during a trial and returns to the screen to initiate a new fixation, the “decision” to look at a certain location is based on the visual information that has been analyzed before, so we do not expect saccade amplitudes to be influenced by this behavior.

There were no differences in the number of fixations made by children with NV and children with VI+nys. An explanation for the absence of a group difference in number of fixations could be the lower percentage of valid data points recorded with the eye-tracker for children with VI+nys. The average percentage of valid data points in a whole session was 62.7% for children with NV and 44.7% for children with VI+nys. An explanation for the low percentage of valid data points in children with VI+nys is that eye movement velocity in these children might often be above 50°/s and above the fixation detection threshold. Characteristics of infantile nystagmus are amplitudes between 0.3 and 15.7°, and frequencies have been reported to range between 0.5 and 10 Hz (Abadi and Bjerre, 2002). Peak velocities of the eye range between 20 and 180°/s (Abadi and Worfolk, 1989). Therefore, less fixations might have been detected in these children, because the algorithm did not find a fixation (i.e., at times when eye movement velocity was above 50°/s). Furthermore, factors such as photophobia, long eye lashes and/or glasses might also cause signal loss during eye-tracking in children with ocular disorders.

### Longer search times for children with VI than children with NV at small spacings

Our second hypothesis was that search times of children with VI+nys are longer than search times of children with NV. We did observe longer search times for children in the VI+nys group than children in the NV group (5.2 s for children in the VI+nys group and 3.7 s for children in the NV group).

The group differences reported in the present study are much smaller than previously reported differences in search time between children with NV and children with VI. For example, in an earlier study where children had to search for a unique target in a grid with elements sized 2 times their threshold acuity, we found up to 5-fold slower search times for children with VI compared to children with NV (Huurneman et al., 2014). Search times for 9–18 year old children with low vision have been reported to be 3-fold compared to that of normally sighted age-matched peers for search in naturalistic wide-field 58 × 45° displays (Tadin et al., 2012). Two explanations might account for the less pronounced group differences in search time in the present study.

A first explanation is that the size of the stimuli was 2 × 2°, which is obviously larger than the average symbol size of 0.67° used in a previous study (Huurneman et al., 2014). Children in the VI+nys group therefore seem to benefit from working with large stimuli. A second explanation might be that the present task is more structured than a naturalistic wide-field display (Tadin et al., 2012). The grid consisted of E's presented in our feature search task might be perceived as a pattern and poses only a little demand on precise eye movements. Some studies indicate that patterns with discriminable elements in close proximity can be segregated more easily than patterns in which the same elements are more widely spaced (Nothdurft, 1985, 1993; Scolari et al., 2007). Deviances, or unique features, within a pattern consisting of homogeneous distractors can be found easily, and this process has been said to be even easier with small element spacing. However, in the present study we observed, for the second time, that during matrix search in children small element spacing does not facilitate search (Huurneman et al., 2014). Search times were unaffected by spacing. The absence of an increase in search time with increasing number of distractors indicates that the search task was conducted in a parallel manner (Treisman and Gelade, 1980).

### Shorter search times in children with VI after training

The third hypothesis was that we expected training to induce shorter search times in all training groups. Children with VI accompanied by nystagmus did show shorter search times after training. Only the two experimental groups showed reduced search times for all spacings. The control PL group did not show a reduction in search time at small spacings. An explanation for the absence of a training effect at small spacings for the PLu group might be that children in this training group did not work with closely spaced elements. In our previous study we also observed differences in the amount of improvement children made with regards to their crowded NVA. All children showed an improvement in single NVA, but only children in the PLc group showed an improvement on the crowded NVA chart (Huurneman et al., 2013).

### Changes in oculomotor measures after training

Our fourth hypothesis was that fixation duration could not be changed by training in children with VI+nys. In contrast to our hypothesis, the results did show changes in fixation duration after training. The children in the PLc group showed prolonged fixation durations at 0.5° (from 242 to 303 ms) and 2° element spacing (from 222 to 294 ms). In addition, the PLu group showed prolonged fixation duration at 1° spacing, but the effect was not observed at other spacings in this training group and seemed to be less consistent than the increase in fixation duration in the PLc group.

The prolongation of fixation durations in the PLc group is an unexpected and exciting finding, because individuals with nystagmus are considered to be unable to voluntary diminish their nystagmus and attempts to prolong fixation periods usually have a negative impact on nystagmus (Sheth et al., 1995). Earlier research has reported the beneficial impact of interventions other than surgery on prolonging fixation duration in individuals with INS, e.g., afferent stimulation by providing vibratory neck stimulation (Sheth et al., 1995). To our knowledge there have not been studies reporting the positive impact of PL on fixation characteristics in individuals with nystagmus. As might be expected from the large body of literature emphasizing the relation between acuity and foveation periods, prolongation of fixation duration indeed was significantly related to a reduction of crowding ratios (*r* = 0.65) and improvements in crowded NVA (*r* = 0.52). In an earlier study, we found that nystagmus frequency was positively related to mono- and binocular crowding ratios in children with idiopathic nystagmus (Huurneman and Boonstra, 2013).

Three explanations may account for changes in fixation duration after training. The first is maturation of the nervous system, which may alter the sinusoidal and jerk nystagmus so that foveation time increases and vision improves (Van Vliet, 1982). A second explanation for the prolonged fixation periods is that increased visual task demand and visual attention can also alter nystagmus movements (Wiggins et al., 2007). When adults with nystagmus were looking at a threshold sized target, they demonstrated longer foveation duration and reduced nystagmus frequency than when looking at a larger target sized 3 log steps above their threshold acuity. This finding indicates that increased visual demand or visual attention may dampen oscillatory eye movements in nystagmus. We think that the observed improvements are probably due to improved visual attentional processing, because during training children worked with small densely packed optotypes which required focused visual attention. A third explanation is that the prolonged fixation durations after training might be due to familiarity with the test display (Cham et al., 2008, 2013). However, reduced stress does not explain the differences in fixation duration gain between training groups. We therefore conclude that more than one explanation might be applicable for the more extended improvements in the experimental PL group.

The mechanism responsible for the extended fixation durations after training is not known, since we only recorded eye movements. A combination of psychophysiological and brain imaging techniques would be required to find out whether changes on a behavioral level have a specific neural origin. One might expect that the reticular formation is involved, a brain structure responsible for arousal, alertness and awareness (Role and kelly, 1991; Sheth et al., 1995), the frontal eye fields, an area responsible for the control of voluntary eye movements (Braddick and Atkinson, 2011) and covert shifts of attention (Ronconi et al., 2012), the superior colliculus (controlling head orientations and adjusting eye movements in response to retinal slip), and/or the cerebellar vermis (Leguire et al., 2011). The presence of ocular torticollis, referring to compensatory head turns made in order to fixate the eyes at the null-point, should also be monitored, because it could also be possible that convergence or gaze angles caused prolonged fixation durations (Abadi, 2002). Future research is needed to investigate the physiological mechanisms underlying extended fixation periods.

## Conclusions

The present study indicates that PL in children with VI showing nystagmus does not only transfer to improvements in NVA, but also induces improvements in feature search performance and oculomotor measures. The most striking finding observed in this study is that children with VI prolonged their fixation duration after training. This prolongation of fixation duration is significantly related with improvements in crowded NVA and reduction of crowding ratios.

### Conflict of interest statement

The authors declare that the research was conducted in the absence of any commercial or financial relationships that could be construed as a potential conflict of interest.
