# Supplementary material for: Training shortens search times in children with visual impairment accompanied by nystagmus
Source: Front Psychol. 2014 Sep 12;5:988. doi: 10.3389/fpsyg.2014.00988 (PMC4162385; doi:10.3389/fpsyg.2014.00988)
Supplement: Supplementary file 1 [file DataSheet1.ZIP › Supplemental Figure 1.PDF]

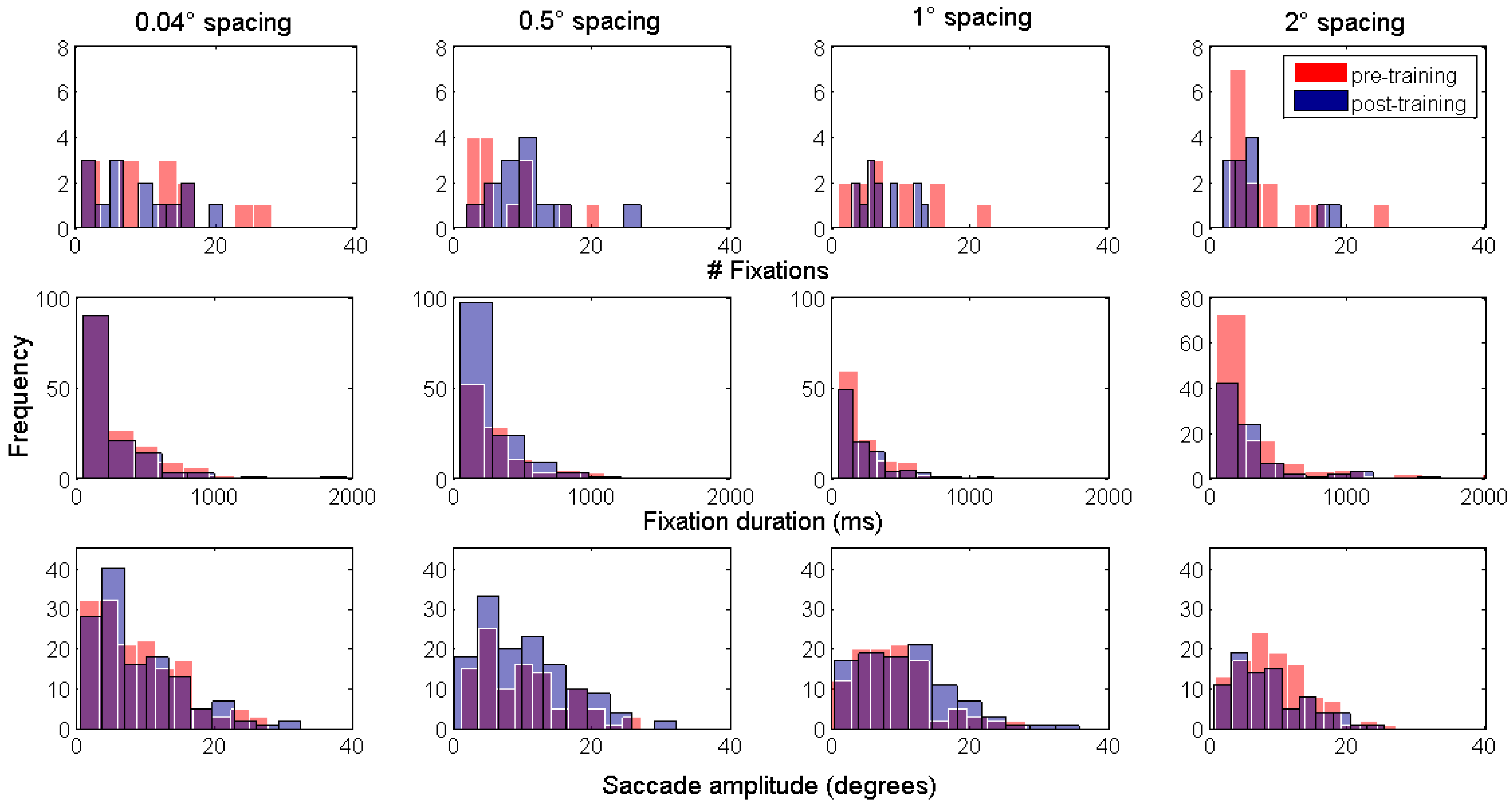

Supplemental Figure 1. The histograms containing all raw pre- and post-training eye movement data for children with VI+nys in the **M group.** Statistical results are presented in the main body of the manuscript. With respect to fixations, children made less fixations at 2° spacing after training (10.0 pre-training and 7.1 post-training; all training groups). Fixation duration and saccade amplitude did not change after training in the M group.
