# Supplementary material for: Training shortens search times in children with visual impairment accompanied by nystagmus
Source: Front Psychol. 2014 Sep 12;5:988. doi: 10.3389/fpsyg.2014.00988 (PMC4162385; doi:10.3389/fpsyg.2014.00988)
Supplement: Supplementary file 1 [file DataSheet1.ZIP › Supplemental Table.DOCX]

| Supplement . Clinical diagnosis and characteristics of children with visual impairment^1^. | | | | | | | | | |
| --- | --- | --- | --- | --- | --- | --- | --- | --- | --- |
| # | **Age (months)** | **Training** | **Clinical diagnosis** | **DVA*** | | | **NVA**** | **Nystagmus** | **Correction** |
|  |  |  |  | **RE** | **LE** | **Bino** |  |  |  |
| 11 | 64 | Magnifier | Oculocutaneous albinism | 1.4 | 1.1 | 0.9 | 1.0 | yes | No correction. |
| 12 | 66 | Magnifier | Aniridia | 1.0 | 1.2 | 0.9 | 0.9 | yes | RE S: +3.75 C: -1.50 ax: 180  LE S: +3.25 C: -2.50 ax: 174 |
| 23 | 61 | Magnifier | Oculocutaneous albinism | 1.1 | 1.0 | 0.9 | 0.9 | yes | RE S: +6.00 C: -1.00 ax: 6  LE S: +5.00 C: -0.75 ax: 6 |
| 26 | 68 | Magnifier | Oculocutaneous albinism | 1.1 | 1.1 | 1.0 | 1.2 | yes | RE S: +2.00 C: -0.50 ax: 180  LE S: +3.75 C: -0.50 ax: 180 |
| 39 | 94 | Magnifier | Ocular albinism | 0.9 | 1.1 | 0.8 | 0.8 | yes | RE S: +0.50 C: -1.25 ax: 105  LE S: plano C: -0.50 ax: 48 |
| 40 | 90 | Magnifier | Congenital nystagmus | 1.0 | 0.7 | 0.6 | 0.6 | yes | RE S: +2.00 C: -1.25 ax: 172  LE S: +2.25 C: -0.50 ax: 6 |
| 44 | 79 | Magnifier | Congenital nystagmus | 0.8 | 1.0 | 0.5 | 0.5 | yes | RE S: +2.00 C: -1.50 ax: 130  LE S: +3.00 C: -1.50 ax: 160 |
| 45 | 75 | Magnifier | Ocular albinism | 1.4 | 1.0 | 1.0 | 1.1 | yes | RE S: +4.25 C: -2.50 ax: 10  LE S: +4.75 C: -2.00 ax: 170 |
| 47 | 95 | Magnifier | Ocular albinism | 1.1 | 1.1 | 0.8 | 0.9 | yes | RE S: +1.75 C: -2.00 ax: 5  LE S: +2.75 C: -2.50 ax: 172 |
| 4 | 74 | PLc | Achromatopsia | 1.0 | 1.0 | 1.0 | 1.0 | yes | RE S: +3.50 C: -3.50 ax: 8  LE S: +3.25 C: -2.50 ax: 174 |
| 7 | 74 | PLc | Oculocutaneous albinism | 1.3 | 1.3 | 1.1 | 1.1 | yes | RE S: +3.50 C: -2.75 ax: 180  LE S: +5.00 C: -2.00 ax: 176 |
| 8 | 74 | PLc | Oculocutaneous albinism | 1.0 | 1.0 | 0.9 | 1.1 | yes | RE S: +6.00 C: -2.50 ax: 3  LE S: +3.25 C: -2.50 ax: 172 |
| 9 | 101 | PLc | Congenital nystagmus | 0.6 | 0.7 | 0.5 | 0.8 | yes | No correction. |
| 15 | 106 | PLc | Oculocutaneous albinism | 1.0 | 1.0 | 1.0 | 1.0 | yes | RE S: +5.25 C: -1.75 ax: 175  LE S: +5.50 C: -3.50 ax: 175 |
| 18 | 108 | PLc | Congenital nystagmus | 0.9 | 0.9 | 0.8 | 0.9 | yes | RE S: +0.75 C: -4.75 ax:5  LE S: +0.75 C: -5.25 ax: 165 |
| 20 | 106 | PLc | Congenital nystagmus | 0.5 | 1.0 | 0.4 | 0.4 | yes | RE S: +0.50 C: -2.50 ax: 14  LE S: +1.00 C: -3.75 ax: 155 |
| 25 | 70 | PLc | Cone dystrophy | 0.3 | 0.3 | 0.3 | 0.5 | yes | RE S: -6.00 C: -1.00 ax: 2  LE S: -6.75 C: -0.75 ax: 50 |
| 28 | 61 | PLc | Ocular albinism | 1.1 | 1.1 | 1.0 | 1.1 | yes | RE S: +4.00  LE S: +4.00 |
| 33 | 57 | PLc | Congenital nystagmus | 0.6 | nm | 0.6 | 0.4 | yes | No correction. |
| 34 | 66 | PLc | Congenital nystagmus | 0.8 | 0.8 | 0.7 | 0.7 | yes | RE S: +2.00 C: -1.00 ax: 180  LE S: +2.00 C: -1.00 ax: 170 |
| 35 | 109 | PLc | Oculocutaneous albinism | 0.5 | 0.6 | 0.4 | 0.4 | yes | RE S: +1.50 C: -2.00 ax: 180  LE S: +1.50 C: -1.25 ax: 175 |
| 37 | 74 | PLc | Retinal dystrophy | 1.1 | 1.0 | 1.0 | 0.8 | yes | RE S: plano C: -1.50 ax: 8  LE S: +0.25 C: -1.50 ax: 166 |
| 42 | 85 | PLc | Achromatopsia | 1.3 | 1.3 | 1.1 | 1.2 | yes | RE S: +4.00 C: -0.75 ax: 180  LE S: +3.00 |
| 48 | 62 | PLc | Congenital nystagmus | 0.8 | 0.7 | 0.7 | 0.8 | yes | RE S: +1.00  LE S: +1.50 |
| 2 | 61 | PLu | Congenital nystagmus | 0.8 | 0.8 | 0.9 | 0.8 | yes | RE S: +0.25 C: -0.75 ax: 166  LE S: +0.50 C: -1.00 ax: 16 |
| 3 | 108 | PLu | Nystagmus | 0.6 | 0.6 | 0.5 | 0.6 | yes | RE S: +3.50 C: -1.50 ax: 10  LE S: +2.50 |
| 5 | 59 | PLu | Aniridia | 1.4 | 1.4 | 1.4 | 1.2 | yes | RE S: -4.75 C: -2.00 ax: 180  LE S: -4.50 C: -1.25 ax: 5 |
| 14 | 104 | PLu | Ocular albinism | 0.9 | 0.9 | 0.9 | 0.9 | yes | RE S: +5.50 C: -3.50 ax: 180  LE S: +5.25 C: -4.50 ax: 180 |
| 16 | 84 | PLu | Oculocutaneous albinism | 1.0 | 1.0 | 0.9 | 0.8 | yes | No correction. |
| 17 | 104 | PLu | Congenital nystagmus | 0.7 | 0.6 | 0.5 | 0.6 | yes | RE S: -2.75  LE S: -2.50 |
| 22 | 80 | PLu | Congenital nystagmus | 0.4 | 0.6 | 0.4 | 0.4 | yes | RE S: +3.00 C: -0.75 ax: 124  LE S: +2.75 C: -1.75 ax: 32 |
| 24 | 95 | PLu | Congenital glaucoma | 0.7 | 0.7 | 0.6 | 0.5 | yes | RE S: -7.50 C: -1.50 ax: 118  LE S: -3.00 C: -0.75 ax: 25 |
| 27 | 95 | PLu | Congenital nystagmus | 0.6 | 0.6 | 0.6 | 0.6 | yes | RE S: +3.25 C: -1.25 ax: 8  LE S: +2.75 C: -1.25 ax: 180 |

^1^ This table presents a subselection of the child characteristics reported in a previous study (Huurneman et al., 2013).

* Distance visual acuity (DVA) measured with C-test, crowded version 2.6′ spacing at 5 m (logMAR notation).

** Near visual acuity measured with the C-test, crowded version 2.6′ at 40 cm (logMAR notation).
